# Water layer and radiation damage effects on the orientation recovery of proteins in single-particle imaging at an X-ray free-electron laser

**DOI:** 10.1038/s41598-023-43298-1

**Published:** 2023-09-29

**Authors:** Juncheng E, Michal Stransky, Zhou Shen, Zoltan Jurek, Carsten Fortmann-Grote, Richard Bean, Robin Santra, Beata Ziaja, Adrian P. Mancuso

**Affiliations:** 1 European XFEL, Holzkoppel 4, 22869 Schenefeld, Germany; 2 Institute of Nuclear Physics, Polish Academy of Sciences, Radzikowskiego 152, 31-342 Kraków, Poland; 3 Max Planck Institute for the Structure and Dynamics of Matter, Luruper Chaussee 149, 22761 Hamburg, Germany; 4 Center for Free-Electron Laser Science, Deutsches Elektronen-Synchrotron DESY, Notkestr. 85, 22607 Hamburg, Germany; 5 The Hamburg Centre for Ultrafast Imaging, Luruper Chaussee 149, 22761 Hamburg, Germany; 6 Department of Physics, Universität Hamburg, Notkestr. 9-11, 22607 Hamburg, Germany; 7 Diamond Light Source, Harwell Science and Innovation Campus, Didcot, Oxfordshire OX11 0DE UK; 8 Department of Chemistry and Physics, La Trobe Institute for Molecular Science, La Trobe University, Melbourne, VIC 3086 Australia

**Keywords:** X-rays, Atomic and molecular interactions with photons, Single-molecule biophysics

## Abstract

The noise caused by sample heterogeneity (including sample solvent) has been identified as one of the determinant factors for a successful X-ray single-particle imaging experiment. It influences both the radiation damage process that occurs during illumination as well as the scattering patterns captured by the detector. Here, we investigate the impact of water layer thickness and radiation damage on orientation recovery from diffraction patterns of the nitrogenase iron protein. Orientation recovery is a critical step for single-particle imaging. It enables to sort a set of diffraction patterns scattered by identical particles placed at unknown orientations and assemble them into a 3D reciprocal space volume. The recovery quality is characterized by a “disconcurrence” metric. Our results show that while a water layer mitigates protein damage, the noise generated by the scattering from it can introduce challenges for orientation recovery and is anticipated to cause problems in the phase retrieval process to extract the desired protein structure. Compared to these disadvantageous effects due to the thick water layer, the effects of radiation damage on the orientation recovery are relatively small. Therefore, minimizing the amount of residual sample solvent should be considered a crucial step in improving the fidelity and resolution of X-ray single-particle imaging experiments.

## Introduction

Single-particle imaging (SPI) experiments have been performed at various X-ray free-electron laser (XFEL) facilities^1–3^, aiming to exploit the XFEL generated pulses to determine the structure of single, non-crystalline biological molecules. This type of experiment takes advantage of the ultrahigh peak brightness and ultra-short pulse duration offered by XFELs, which results in continuous improvement of the resolution^4–7^. However, the target resolution, at length scales of a few Ångströms ($10^{-10}$ m)^8^, has not yet been achieved^9^.

Several issues posing significant challenges to achieve higher spatial resolution in SPI experiments, such as sample heterogeneity (including sample solvent)^10,11^, sample delivery^12^, detector noise^13^ and radiation damage^14,15^, have been identified^16^. The issues of the sample solvent and radiation damage are interdependent, as the sample solvent can influence the radiation damage of a protein sample and, thus, its effect on the resulting diffraction pattern. On the one hand, the solvent acts as a tamper layer slowing down the expansion of the protein that occurs due to ionization of its component atoms^17^. On the other hand, the solvent itself is a source of background scattering noise^10^. Water is a common solvent for bio-molecules and is expected to remain attached as a thin layer around the sample even after most of it has evaporated when using the electrospray technique to deliver the sample to an X-ray beam^11,18^. The impact of water layer thickness on SPI experiments has been previously investigated by evaluating the features of diffraction patterns from hydrated single particles^11,15,19,20^. However, the combined effect of the radiation damage and water layer thickness on the complete orientation recovery process^21^ was not fully addressed in the previous studies.

Orientation recovery aims to sort a set of diffraction patterns that have been generated by scattering from identical particles placed at unknown orientations. The goal of an orientation recovery algorithm is to find to which orientation each diffraction pattern belongs, with the ultimate aim of assembling a three-dimensional (3D) reciprocal space volume which can be inverted (“phase retrieved”) to yield the 3D real-space structure of the particle under investigation. The quality of the orientation recovery can significantly affect the result of the phase-retrieval analysis, particularly, when the scattering signal from single particles is low (as for biomolecules). The expand-maximize-compress (EMC) algorithm^4,22–24^ is the most commonly used orientation recovery method for SPI experiments at XFEL facilities.

In order to estimate the spatial resolution of an SPI experiment, several methods are proposed^25^. Fourier Shell Correlation (FSC) is one such method and is widely employed for SPI data analysis^4,7,26–28^. Although FSC is widely used for analyzing reconstruction results, as pointed out by Shen et al.^29^, a less precise (and hence “more blurred”) orientation recovery can actually lead to an illusory enhancement in FSC. The reason is that this blurring effect attenuates noise originating from diffraction patterns and errors in orientation determination, resulting in an apparent (though misleading) similarity among reconstructions.

In our case, a challenge is that the particles are surrounded by a layer of solvent, which manifests in the reciprocal space volume with a blurring effect. This blurring effect can then hinder the orientation determination process. Thus we have adopted the “orientation disconcurrence” (OD) metric^29^ to assess the quality of the reconstructed reciprocal space volume. This method directly estimates the orientation-decoding ability of a diffraction volume for a given set of patterns, which provides a more universal and less ambiguous way to evaluate the quality of orientation recovery. The details of the metric are described in the Methods section.

Continuing from our previous work^15^, we investigate the impact of radiation damage and water layer thickness in hydrated proteins on the orientation recovery process. In particular, we study their effects on the reciprocal space volume reconstructed from (previously) simulated diffraction patterns of the same particle surrounded by a water layer in a variety of orientations^15,30^, quantify these effects with the OD metric^29^, and analyze the causes for the observed changes in the OD for various water layer thicknesses.

## Orientation recovery of hydrated 2NIP protein

The simulation of diffraction data proceeded as described in detail in Ref. ^15^. The nitrogenase iron protein (PDB: 2NIP) was covered with water layers of varying thickness (0, 2, 4, 6, 10 and 20 Å) and the simulation was run with and without radiation damage taken into account.

The radiation damage followed the impact of 55 different simulated X-ray SASE pulses of 4.96 keV photon energy and a full duration at half maximum (FDHM) of 9 fs. Each pulse had approximately $7.5 \times 10^{12}$ photons after propagation through the X-ray optics. The nominal focus size was $250 \times 160$ nm$^2$ FWHM, yielding a fluence of $1.5 \times 10^7$ J/cm$^2$ and an intensity of $1.6 \times 10^{21}$ W/cm$^2$.

For each water layer thickness, we generated 20000 diffraction patterns with different orientations, incident wavefronts, and water-protein conformations (55 incident wavefronts and 125 water-protein conformations were utilized repeatedly). The diffraction datasets for different water layer thicknesses all followed the same uniform distribution over the SO(3) rotation group.

In order to make the simulations more realistic, Poisson noise was applied to the diffraction patterns. The average number of photons per pattern was around 500. Typical diffraction patterns with different water layer thicknesses are shown in Fig. S1 in the supplementary material. The details of diffraction pattern generation are in the Methods section.

The *Dragonfly* software package (Version 1.2.0)^23^ was implemented using the EMC algorithm^22^ to recover the 3D orientation of our simulated 2D diffraction patterns. For details on the reconstruction parameters, see the Methods section.

We define the ground truth for a given water layer thickness as the ideal reciprocal space intensity distribution calculated directly from the undamaged sample with a specific water layer thickness, in a fixed water–protein configuration.

For each layer thickness and radiation damage condition, we reconstructed the reciprocal space intensity distribution from the 20000 diffraction patterns with Poisson noises and aligned them to the ground truth reference.

**Figure 1:**
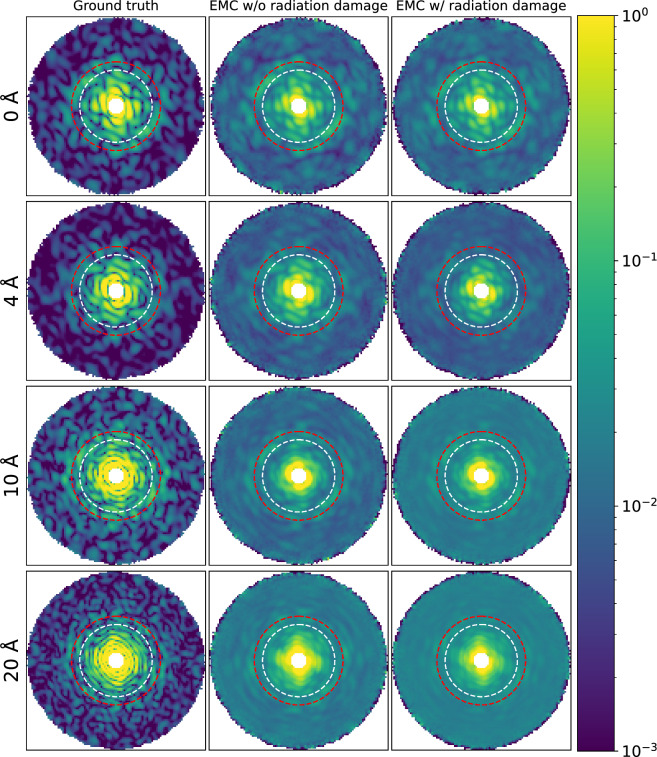
Central XY slices of the ground truth reciprocal space volumes and the reciprocal space volumes reconstructed from diffraction patterns with and without radiation damage, and with varying water layer thicknesses. The white and red dashed circles are at $q =$ 0.08 Å$^{-1}$ and $q =$ 0.10 Å$^{-1}$, respectively. The *q* value at the edge is 0.2 Å$^{-1}$. The color map representing intensity is plotted in arbitrary units.

Figure 1 displays XY slices taken from each of the reconstructed reciprocal space intensity distributions under the aforementioned conditions with a *q* value of 0.2 Å$^{-1}$ at the edge of the slice. Here, the scattering vector length *q* is defined as $q = 2 \sin \theta / \lambda$, where $\theta$ is half the diffraction angle and $\lambda$ is the X-ray wavelength. Both elastic and inelastic scattering are considered in the input diffraction patterns. For cases showing only the elastic scattering, please see the supplementary material (Figs. S3–S4). The slices in reciprocal space show that the speckles in the high-*q* region ($q \ge$ 0.1 Å$^{-1}$) are barely distinguishable from the background noise, while at the low-*q* region ($q<$ 0.08 Å$^{-1}$), the feature can be approximately reconstructed, if the water layer thickness is below 10 Å. In contrast, when the water layer is thicker than 10 Å, even the features in the low-*q* region are smeared out.

**Figure 2:**
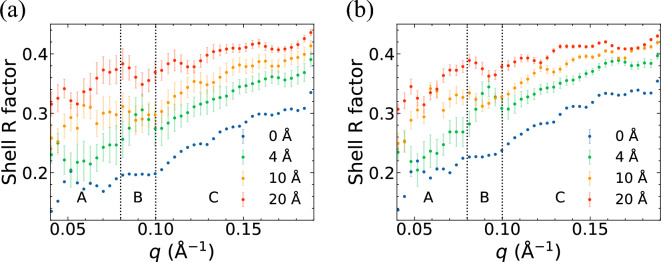
The shell R factor averaged over 3 independent EMC reconstructions from each diffraction dataset (**a**) without radiation damage and (**b**) with radiation damage. The error bars are the standard deviations of the shell R factors of the 3 reconstructions. The two vertical dashed lines are at $q =$ 0.08 Å$^{-1}$ and $q =$ 0.10 Å$^{-1}$, respectively.

Those details can be revealed more clearly by the shell R-factor to quantify local orientation recovery qualities. Derived from the conventional R-factor^13,31^ used for resolution estimation , the shell R-factor of a given reciprocal space shell (*s*) is:

1 $$\begin{aligned} R(s) = \sum _{{\textbf{q}} \in s}{\mid }\frac{\sqrt{I({\textbf{q}})}}{\sum _{{\textbf{q}}' \in s}\sqrt{I({\textbf{q}}')}} - \frac{\sqrt{I_{ideal}({\textbf{q}})}}{\sum _{{\textbf{q}}' \in s}\sqrt{I_{ideal}({\textbf{q}}')})}{\mid }, \end{aligned}$$

where $I({\textbf{q}})$ is the intensity at a scattering vector ${\textbf{q}}$, and $I_{ideal}({\textbf{q}})$ is the intensity from the reference (undamaged) sample without any additional noise. In Fig. 2, the shell R factor shows different characteristics of region B (0.08 $\le$ *q* < 0.1 Å$^{-1}$, where *q* is the magnitude of ${\textbf{q}}$) for different water layer thicknesses. In that region, it is flat for the reconstructed volume without a water layer; with water layer thickness $T_w$ = 4 Å, it increases and then decreases, while it decreases and then increases with $T_w \ge 10$ Å. In the low-*q* region A (*q* < 0.08 Å$^{-1}$), the mean shell R factor value (${\bar{R}}$ and $\bar{R'}$) ranges from 0.19 to 0.26 (Table 1) for $T_w$ below 4 Å, where the speckles are still recognizable in Fig. 1. In the same region, ${\bar{R}}$ and $\bar{R'}$ reach above 0.3, and some fringes are already lost in the reconstruction for $T_w$ above 10 Å (Fig. 1). In the high-*q* region C ($q \ge$ 0.1 Å$^{-1}$), ${\bar{R}}$ and $\bar{R'}$ are above 0.41. With the high shell R factor there, the speckles appear significantly blurred in the high-*q* region C of Fig. 1. The standard deviation of the shell R factor without radiation damage is higher than that with radiation damage. The lower variance with radiation damage is attributed to blurrier diffraction patterns affected by radiation damage. Orientation recovery from blurrier diffraction patterns is easier to converge but does not necessarily lead to a better reconstruction quality^29^.

**Table 1:** Values of the mean shell R factor from reconstructed reciprocal space volumes obtained for various water layer thickness ($T_w$) without radiation damage (${\bar{R}}$) and with radiation damage ($\bar{R'}$) in the regions A: *q* < 0.08 Å$^{-1}$, B: 0.08 $\le$ *q* < 0.1 Å$^{-1}$, and C: $q \ge$ 0.1 Å$^{-1}$ of Fig 2.

$T_w$	${\bar{R}}_A$	${\bar{R}}_B$	${\bar{R}}_C$	$\bar{R'}_A$	$\bar{R'}_B$	$\bar{R'}_C$
0 Å	0.19	0.20	0.42	0.21	0.23	0.41
4 Å	0.25	0.28	0.46	0.26	0.32	0.51
10 Å	0.30	0.30	0.50	0.31	0.32	0.52
20 Å	0.33	0.37	0.54	0.33	0.38	0.53

## Orientation disconcurrence analysis

We employed the self-OD ($\Delta \theta$) analysis^29^ to evaluate the quality of orientation reconstruction. This metric quantifies the uncertainty of the orientation recovery for patterns in reconstructed reciprocal space intensity volumes. For its mathematical definition, please see the Methods section. For each water layer thickness, we calculated the self-OD for all the 3 reciprocal space volumes reconstructed in the previous section. For each reconstruction, 500 diffraction patterns (that were calculated in the same condition as the reconstruction input diffraction patterns but not used for reconstruction) were utilized as a reference (sentinel patterns) for the self-OD analysis. The larger $\Delta \theta$ indicates a larger uncertainty, which is mainly attributed to worse reconstruction and distribution change in diffraction intensities. For further details, please refer to the Methods section and the OD paper^29^.

**Figure 3:**
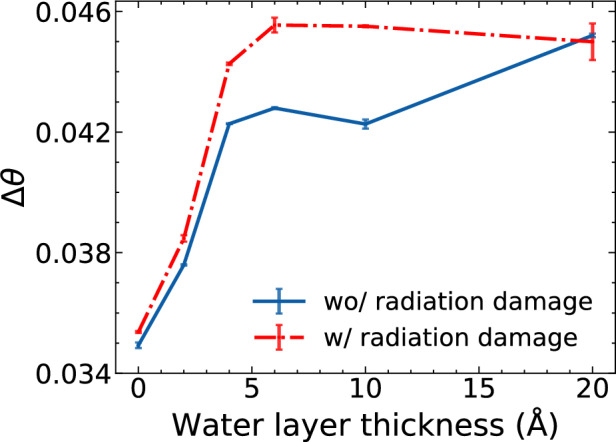
The self-orientation-disconcurrence of the orientation recovery with/without radiation damage as a function of water layer thicknesses. Each point on the curves is averaged over 3 independently reconstructed reciprocal space volumes, and its error bar is the standard deviation from the mean $\Delta \theta$. A lower value of $\Delta \theta$ indicates a better orientation recovery quality.

As shown in Fig. 3, we observe that $\Delta \theta$ increases as the water layer thickness increases. Compared with the $\Delta \theta = 0.027$ of the ground truth without a water layer and radiation damage, the $\Delta \theta$ values of the reconstructions are higher due to the reconstruction quality impaired by radiation damage and water layers. In both cases, with and without radiation damage, the increase is steep with $T_w$ less than $\sim 5$ Å and then becomes flat with $T_w$ larger than $\sim 5$ Å. For the cases of $T_w = 20$ Å, the $\Delta \theta$ with radiation damage even decreases slightly, to a value similar to that without radiation damage.

The change in the curve slope could be related to the volume ratio between the protein and the water layer. Once the volume of the water layer surpasses that of the protein ($T_w \ge 5$ Å), the overall shape of the water layer becomes the dominant factor in determining the structure diffraction volume. As the thickness increases further, the shape changes more gradually, leading us to believe that the ability to determine orientation is less affected. The slight dip at ($T_w = 20$ Å) could be due to an asymmetry introduced by a shape change of the thick water layer, which eases the process of orientation determination.

The self-OD intrinsically provides a measure of the uncertainty in reconstructing the orientation of a sentinel diffraction pattern within a given intensity volume (its higher value corresponds to higher uncertainty), thus indicating the quality of the reconstruction. These results suggest that thicker water layers pose greater challenges for orientation reconstruction, and that radiation damage only has a minor effect on recovering orientation.

## Effects of radiation damage on the orientation recovery

Despite the relatively small difference in the self-OD values between the reconstructed reciprocal space with and without radiation damage, it is still beneficial to investigate the impact of radiation damage on the diffraction patterns and the subsequent reconstruction process. While our previous paper^15^ explored the effect of radiation damage on diffraction patterns, it did not address the question of how the patterns become blurred due to radiation damage and how this ultimately affects the orientation recovery process.

**Figure 4:**
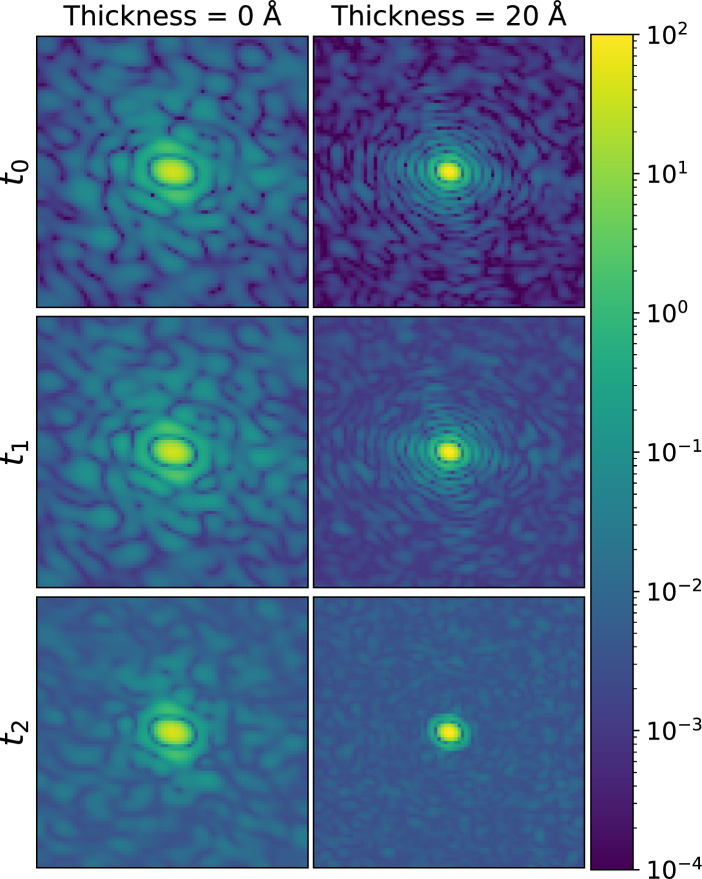
Diffraction patterns (displayed without Poisson noise) with radiation damage taken in the same sample orientation at various time instants ($t_0$ = -10.94 fs, $t_1$ = 2.07 fs, and $t_2$ = 12.47 fs) of the Pulse 1 in Fig. 5, with water layer thickness of 0 Å and 20 Å, respectively. The color map represents intensity. *q* = 0.14 Å$^{-1}$ at the edge of the diffraction patterns. Patterns are normalized to the same mean number of photons (plotted in arbitrary units).

**Figure 5:**
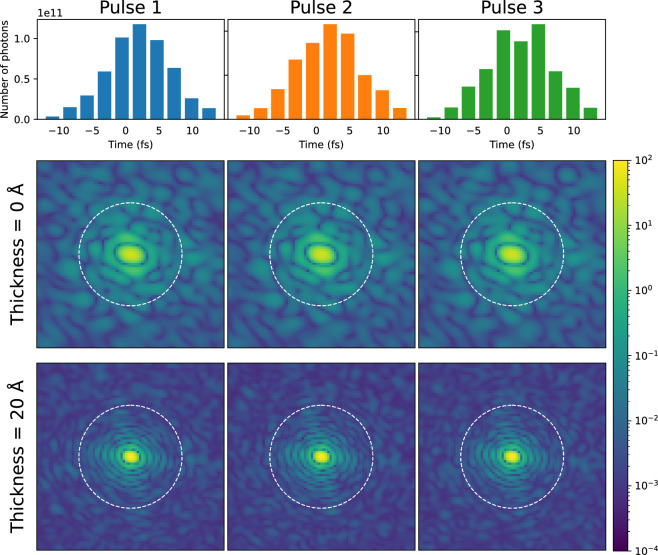
First row: three temporal profiles out of the fifty-five simulated SASE pulses used for diffraction calculation. Each of them is binned with an interval of 2.6 fs. Second and third row: Time-integrated diffraction patterns (displayed without Poisson noise) obtained for the corresponding SASE pulses in the first row. The variance of intensity distribution for different pulses is noticeable at $q \ge$ 0.08 Å$^{-1}$ with a 20–Å water layer, while there is no obvious difference among the pulses at *q* < 0.08 Å$^{-1}$ in the cases of both water layer thicknesses. *q* = 0.14 Å$^{-1}$ at the edge of the diffraction patterns. The white dashed circle is at *q* = 0.08 Å$^{-1}$. The color map represents intensity and is plotted in arbitrary units.

In order to investigate how the diffraction patterns are affected by radiation damage, we evaluated the diffraction patterns from the protein with no water layer and from the protein with water layer thickness $T_w$ = 20 Å. As the number of photons is too low to clearly see the change in the diffraction pattern with Poisson noise included, the patterns in Fig. 4 and Fig. 5 are displayed without Poisson noise. At time point $t_2$, the speckles contract toward the center of the diffraction pattern of both water layer conditions, which reflects that the sample is strongly expanded at the late stage of photon-matter interaction (the process can be clearly observed in the movies uploaded as Supplementary Information 2–3). When *T*_*w*_ = 20 Å, the fringe around the center disappears at *t*_*2*_, but not when *T*_*w*_= 0 Å. It is worth mentioning that in the case with the 20–Å–thick water layer, the expansion affects mostly the water layer, i.e., the protein is much less expanded than in the case without the water layer^15^. 

Due to the fact that each diffraction pattern recorded on a detector is time-integrated over the whole X-ray pulse duration, a change in the transient patterns can blur the final diffraction pattern. The extent of blurring depends on the temporal intensity profile of the X-ray pulse (Fig. 5). While the region of *q* < 0.08 Å$^{-1}$ remains approximately unchanged for both water layer thickness cases, the region of $q \ge$ 0.08 Å$^{-1}$ shows significant differences in intensity distribution when $T_w$ = 20 Å.

## Discussion

The reconstructed reciprocal space volume for cases with $T_w$ = 0 Å and 20 Å in Fig. 1 significantly differ: whilst the former shows the structural features of the 2NIP protein, the latter shows almost no features of the protein but those of a round water droplet (i.e. the rings close to each other near the center of the reciprocal space). The results of the shell R factor in Fig. 2 and the self-OD in Fig. 3 show a deterioration in orientation recovery caused by the water layers: the values of the shell R factor and self-OD increase as the water layer thickness increases. Radiation damage also contributes to the deterioration, but in a very modest extent: the self-OD has increased 30% as $T_w$ increases from 0 to 20 Å without radiation damage, while the increase of self-OD caused by radiation damage is only 1.3% at $T_w = 0$. The results are also consistent with Ref. ^11^. In the paper, Mandl et al. performed MD simulations of ubiquitin and lysozyme with varying levels of hydration at different temperature points. 50 structure trajectories and their 2D diffraction patterns were simulated for each type of the protein. The Fourier ring correlation (FRC) analysis of the diffraction patterns reveals that, although a water layer of $T_w = 6$ Å can help with bringing heterogeneous proteins close to one common mean structure, the benefit is counteracted by the scattering noise of the additional water.

If extraction of the protein-only diffraction pattern by subtracting the water-only background diffraction pattern from the hydrated protein diffraction pattern would be possible, this could be an alternative solution to this problem. However, given that the contribution of the water background also differs from shot-to-shot in the experiment, it is unclear how to subtract the correct diffraction pattern. Also, there is presently no reliable way to control the thickness of the residual water layer. The water layer structure is assumed to not be the same from particle to particle, except perhaps in the regions nearest the particle where it may be most influenced by the structure of the protein. In practice, the diffraction patterns collected in an SPI experiment can come from a mixture of various water layer thicknesses , adding further difficulty to structure reconstruction: the varying shape around the diffraction central speckle can confuse not only the EMC algorithm but also other structural analyses presuming all particles have the same conformation.

If the amount of residual water around the sample is reduced, radiation damage becomes the main detriment to diffraction pattern quality (although not as predominant as the water layer). Fig. 4 shows the diffraction patterns at certain time points ($t_0$–$t_2$) with different water layer thicknesses ($T_w$ = 0 Å and 20 Å). In the case of $T_w$ = 20 Å, there is a clear difference between the diffraction patterns at the time points $t_1 = 2.07$ fs and $t_2 = 12.47$ fs: after the maximum of the X-ray pulse, the fringes disappear and the speckles become dense and small. In the case of $T_w$ = 0 Å, the respective changes of the diffraction patterns are still noticeable, though less pronounced. The difference in the effect of radiation damage for “dry” and hydrated protein cases can be attributed to the faster Coulomb explosion facilitated by enhanced secondary ionization^15^.

Despite observing strong radiation damage at the end of an XFEL SASE pulse, due to its ultra-short pulse length (the first row in Fig. 4), the diffraction signal from the strongly damaged structure (within the 2.6–fs interval around $t_2$) only contributes to 0.9% (see Fig. S2 in the supplemental material) of the diffraction intensity registered by a detector. As a result, the diffraction pattern can still be well-preserved.

The results are also supported by similar findings obtained with different simulation methods^32–34^. Martin et al.^33^ discussed the ‘self-gating’ pulse effect for SPI with a well-established rate-equations model describing the radiation damage and its influence on the diffraction contrast for GroEL protein. In the simulation, the displacement of the ions in the inner part of the GroEL protein is less than 3 Å, and the ions in the outer layer move only after 10 fs. The phenomenon and scale of the displacement match the results in this paper and Ref. ^15^ precisely. By analyzing the signal-to-noise ratio (SNR) of diffraction with radiation damage, the authors concluded that “the damage noise introduced by uncorrelated damage processes is much less than shot noise”. The conclusion is further enhanced later by MD simulation considering the Coulomb explosion of the sample^35^. It shows that the Pearson correlation coefficient for radiation damage is higher than that for sample heterogeneity, and the contribution to the total noise of damage noise is also lower than that of shot noise, indicating a relatively low impact of the radiation damage. These studies with different methods consistently suggest that radiation damage may not be as dominant as sample heterogeneity in affecting SPI resolution.

In Fig. 5, we can see that the speckle features within the entire diffraction pattern are still intact when $T_w$ = 0 Å, while significant variation from pulse to pulse in the region of $q \ge$ 0.08 Å$^{-1}$ is observed when $T_w$ = 20 Å. That change then blurs the reconstruction of the reciprocal space volume and contributes to the difference between the $\Delta \theta$ in the cases with and without radiation damage.

## Conclusions

We have investigated the effect of water layer thickness and radiation damage on the quality of the reconstructed reciprocal space intensity distribution from the diffraction patterns obtained in our previously modeled single-particle imaging experiment^15^ on a sample of the size around 10 nm.

Considering diffraction patterns obtained from “dry” and hydrated protein with varying water layer thickness, and with and without radiation damage, we reconstructed the reciprocal space volume with the EMC algorithm, evaluated the reconstruction quality with the OD metric, and investigated how the radiation damage affects the orientation recovery process for different water layer thicknesses.

The OD curve as a function of water layer thickness suggests that both radiation damage and noise from the sample solvent can impede the orientation recovery. However, the noise from the sample solvent has a more substantial impact than radiation damage. As the water layer thickness increases, the quality of the reciprocal space reconstruction decreases and the deterioration caused by the radiation damage (mostly of the water layer) gets intensified.

Due to the ultra-short duration of the XFEL pulses, the effect of radiation damage in the case without a water layer is small. Therefore, one should consider reducing the water layer thickness as much as possible to suppress the strong effect of the diffraction noise introduced by the X-ray scattering off the water. We also found studies suggesting that residual water enhances the structural stability of a protein. In an ideal case, the thickness of the water layer should be chosen so as to only to stabilize the protein. According to Marklund et al.^18^, a thickness of 3 Å is sufficient to stabilize the proteins under investigation. Considering the difficulty to control the amount of residual water with currently available sample delivery techniques, and the relatively low signal-to-noise ratio for protein in XFEL experiments, we suggest using a sample delivery method that can generate the thinnest water layers on sample and the least background in general. If, in future, a system with a more precise sample environment control, including well-defined and “thin” water layers, is developed, this may provide an alternate route to optimal orientation and phase retrieval in XFEL single-particle imaging.

## Methods

### Diffraction simulation

The simulation parameters of the X-ray beam (4.96 keV photon energy, 9 fs FDHM, $\sim 7.5 \times 10^{12}$ photons/pulse, nominal focus size $250 \times 160$ nm$^2$ FWHM, yielding the fluence of $1.5 \times 10^7$ J/cm$^2$ and the average intensity of $1.6 \times 10^{21}$ W/cm$^2$) were taken based on the design values^36^ of the single particles, clusters, and biomolecules & serial femtosecond crystallography (SPB/SFX) instrument^37^. Considering a nano focus $180 \times 180~\textrm{nm}^2$ with 75% optical efficiency for an X-ray pulse energy of 4 mJ in a real experiment, the slightly larger pulse energy and focus size in our simulation still reflect the current performance of the European XFEL. The photon energy of 4.96 keV was inherited from the previous work^14,15^ for consistency. 55 propagated beam profile instances were generated with the aforementioned beam simulation parameters.

In order to generate real space atomic arrangements representing the protein surrounded by a realization of water layer, we used the methodology discussed by Refs. ^15,38^. With this method, 125 atomic configurations were generated for each water thickness case, serving as the initial configuration for the radiation damage simulations.

We conducted 1000 molecular dynamics (MD) simulation runs with the XMDYN simulation code^39^ for each water layer thickness case^15^. For an MD trajectory, a unique combination of one of the 55 propagated beam profiles and one of the 125 water-protein configurations was used. Finally, for each of the 1000 XMDYN time-resolved trajectories, we took 20 time-integrated diffraction patterns in different orientations. This way, all together, 20000 diffraction patterns were generated for each sample condition (with/without radiation damage, with/without water layer thicknesses). They were calculated with the same list of orientations covering the SO(3) rotation group at the detector geometry of an $81 \times 81$ pixel array. The detector had a pixel size of 1200 $\mathrm{\mu }$m and a sample-to-detector distance of 13 cm. The full-period resolution at the detector edge was approximately 7 Å ($q = 0.14$ Å$^{-1}$). The detector geometry here represents the region of the central $480 \times 480$ pixels of the mega-pixel AGIPD detector^40^ deployed at SPB/SFX. The diffraction patterns were simulated with inelastic scattering and Poisson noise to be as close to the realistic signal as possible, and then processed with the Dragonfly package for orientation recovery.

### Orientation recovery

The orientation recovery from the 2D diffraction patterns to reconstruct the 3D reciprocal space volume was done using the Dragonfly package^23^, which is based on the expand-maximize-compress (EMC) algorithm^22^. It (i) starts from a guessed distribution of random reciprocal space volume intensities, (ii) exports/expands the model into tomograms, (iii) uses expectation-maximization to cluster diffraction patterns into these tomograms, and compresses these “maximized” tomograms into a new model, and (iv) repeats the expand-miximize-compress step until the model converges.

There are two critical parameters adjusted during the orientation recovery process: beta and num_div. beta is a regularization factor to adapt to different peak widths of orientation probability distributions. For the diffraction patterns with a very high signal, a low beta value needs to be set until the reconstruction converges to the neighborhood of the true solution. num_div defines the number of rotations available in a reconstruction’s space, i.e. the level of refinement of the reconstruction^23^. For each set of diffraction patterns, we start with beta = 0.01 and num_div = 5, then increase the value of |beta| by 1.5 times and num_div by 1, simultaneously in every 10 iterations until 120 iterations are reached. In the end, we run the reconstruction for extra 10 iterations with beta = 1, and num_div = 16 corresponding to 204960 rotation samples, into which the diffraction patterns are sorted.

### Orientation disconcurrence

This paper employs the orientation disconcurrence (OD)^29^ as a metric to assess the accuracy of reconstructed reciprocal space volumes. Uncovering the latent orientation of diffraction patterns constitutes a critical step in the reconstruction process, and it profoundly influences the quality of the final real space reconstruction. Therefore, the precision of orientation determination is indicative of the reconstruction quality. The OD quantifies the disagreement in determining the orientations of a set of patterns on two different reconstructed reciprocal space volumes ($W_A$, $W_B$). To ensure generalizability and prevent overfitting, this set of patterns, referred to as “sentinel” patterns, should not be used for reconstruction.

To evaluate the accuracy of reconstructed reciprocal space volumes in this study, we define the uncovered orientations of a sentinel pattern with respect to the two reciprocal space volumes as $\Omega _A$ and $\Omega _B$, respectively, where $W_A$ and $W_B$ can be the same or different. In the presence of an overall orientation between $W_A$ and $W_B$, the OD can be characterized by the variation of the overall orientation, which is calculated as $\Omega _A \Omega _B^{-1}$. After taking care of the overall orientation, we obtain a more comprehensive view of the reconstructions from different orientations by averaging this variation over the entire set of sentinel patterns.

It is important to note that different choices of $(W_A, W_B)$ can emphasize different meanings. For example, if the two reconstructions are from two separate datasets, the corresponding OD can then cover various factors, such as an imperfect reconstruction algorithm, an insufficient dataset, or a low flux beam. More combinations of different kinds of $W_A$ and $W_B$ are discussed in the OD paper^29^. However, to make our discussion concise, in this study, we use the self-OD where $W_A=W_B$. It to some extent overlooks the errors introduced by the algorithm or dataset, thus more directly reflects the impact brought about by the water layer — how it changes the distribution of the reciprocal space intensities then affects the determination of the orientations.

To establish the complete formulas for OD, uncovered orientations, $\Omega$, of a collected pattern, *K*, against a reciprocal space volume, *W*, should be described by a probability, $p(\Omega \mid K; W)$^22^. The scattering of photons of an object gives us the probability of generating *K* at $\Omega$:

2 $$\begin{aligned} p\left( K \mid \Omega ; W\right) = \prod _{t \in \text{ detector } } \frac{\textrm{e}^{-W_{\Omega t}}W_{\Omega t} ^{K_{t}}}{K_{t} !} \text {,} \end{aligned}$$

where $W_{\Omega t}$ is the intensity integrated over a pixel *t* on the Ewald’s sphere rotated by $\Omega$. Since we do not have any a prior knowledge about the orientation, we have

3 $$\begin{aligned} p(\Omega \mid K; W)=\frac{p(K\mid \Omega ; W)}{\int p(K\mid \Omega ; W)\,\textrm{d}\Omega }\text {.} \end{aligned}$$

Then the variation will be in the form of

4 $$\begin{aligned} \Theta ^2(K; W_A, W_B)=\iint _{\Omega _\alpha ,\Omega _\beta \in SO(3)} \theta ^2(\Omega _\alpha , \Omega _\beta )p(\Omega _\alpha \mid K; W_A)p(\Omega _\beta \mid K; W_B)\, \textrm{d}\Omega _\alpha \textrm{d}\Omega _\beta \text {,} \end{aligned}$$

where $\theta (\Omega _\alpha , \Omega _\beta )$ is the rotation angle from $\Omega _\alpha$ to $\Omega _\beta$ and can be regarded as the geodesics distance on the *SO*(3). However, Eq. 4 has a few challenges that need to be addressed.

Firstly, as previously mentioned, the overall orientation $\Omega _A\Omega _B^{-1}$ between $W_A$ and $W_B$ is unknown though affects the variation determination. Since the probability, $p(\Omega \mid K; W)$ only depends on the relative orientation between *K* and *W*, rotating the *W* is equivalent to fixing *W* but rotating *K*. We split the $\Omega _\iota$ ($\iota =\alpha ,\beta$) into $\Omega _\iota \Omega _I$ ($I=A, B$) where the intensity orientation $\Omega _I$ will be determined later by minimizing $\Theta$.

In addition, the symmetry of diffraction intensities can increase the level of variation, rendering the metric useless for assessing quality. For instance, if a sentinel pattern is fitted against a symmetric intensity $W_A$ at a specific orientation $\Omega _A$, it must also be fitted at another orientation $\Omega _s\Omega _A$, where $\Omega _s$ is an orientation in the point group of $W_A$. Although $\Omega _A$ and $\Omega _s\Omega _A$ are equivalent in determining orientation, they yield completely different $\theta$ values when compared to another orientation $\Omega _B$, particularly when $\Omega _A$ and $\Omega _B$ are close. Similarly, if the Ewald’s sphere is flat, the Friedel symmetry will have a similar effect. To solve this problem, a new distance (rotation angle) function is used^41^:

5 $$\begin{aligned} \theta _{z, s}(\Omega _\alpha , \Omega _\beta ) \equiv \min _{\Omega \in s, \Omega ' \in z} \theta (\Omega \Omega _\alpha \Omega ', \Omega _\beta )\text {.} \end{aligned}$$

Here, *s* refers to the known point group of the objects ($s=C_2$ in this paper), and *z* contains the identity orientation and the rotation about the beam direction (the *z* axis in our convention) by $\pi$. The group *z* takes care of the Friedel symmetry. In this way, we quotient out the equivalence relation defined by *s* and *z* on *SO*(3). The new function, $\theta _{s, z}$, is the new geodesic on the new quotient space.

Combining the two patches discussed earlier, we can get the expression of OD, $\Delta \theta$, as follows:

6 $$\begin{aligned} \Delta \theta (W_A, W_B)&= \min _{\Omega _A, \Omega _B} \sqrt{\Bigl \langle \Theta ^2(K; W_A, W_B)\Bigr \rangle _{K\in \text {sentinel}}} \end{aligned}$$

7 $$\begin{aligned} \Theta ^2(K; W_A, W_B)&= \iint _{\Omega _\alpha ,\Omega _\beta \in SO(3)} \theta _{z,s}^2(\Omega _\alpha , \Omega _\beta )\, \textrm{d}\mu (\alpha , A)\,\textrm{d}\mu (\beta , B) \end{aligned}$$

8 $$\begin{aligned} \textrm{d}\mu (\iota , I)&\equiv p(\Omega _\iota \Omega _I \mid K; W_I)\, \textrm{d}\Omega _\iota \end{aligned}$$

And the self-OD, $\Delta \theta (W)$ is the special case when $W_A=W_B=W$ in Eq. 6.

### Supplementary Information

Supplementary Information 1.

Supplementary Information 2.

Supplementary Information 3.

## Data Availability

Data are available from the corresponding author upon reasonable request.
