# Supplementary material for: Water layer and radiation damage effects on the orientation recovery of proteins in single-particle imaging at an X-ray free-electron laser
Source: Sci Rep. 2023 Sep 29;13:16359. doi: 10.1038/s41598-023-43298-1 (PMC10541445; doi:10.1038/s41598-023-43298-1)
Supplement: Supplementary file 1 — Supplementary Information 1. [file 41598_2023_43298_MOESM1_ESM.pdf]

Supplementary material: Water layer and radiation damage effects on the orientation recovery of proteins in single-particle imaging at an X-ray Free-Electron Laser

Juncheng E[1*], Michal Stransky[1,2†], Zhou Shen[3], Zoltan Jurek[4,5], Carsten Fortmann-Grote[1], Richard Bean[1], Robin Santra[4,5,6], Beata Ziaja[4,2], and Adrian P. Mancuso[1,7,8*]

[1]European XFEL, Holzkoppel 4, 22869 Schenefeld, Germany
[2]Institute of Nuclear Physics, Polish Academy of Sciences, Radzikowskiego 152, 31-342, Krakow, Poland
[3]Max Planck Institute for the Structure and Dynamics of Matter, Luruper Chaussee 149, 22761, Hamburg, Germany
[4]Center for Free-Electron Laser Science, Deutsches Elektronen-Synchrotron DESY, Notkestr. 85, 22607 Hamburg, Germany
[5]The Hamburg Centre for Ultrafast Imaging, Luruper Chaussee 149, 22761 Hamburg, Germany
[6]Department of Physics, Universität Hamburg, Notkestr. 9-11, 22607 Hamburg, Germany
[7]Diamond Light Source, Harwell Science and Innovation Campus, Didcot, Oxfordshire OX11 0DE, UK
[8]Department of Chemistry and Physics, La Trobe Institute for Molecular Science, La Trobe University, Melbourne, VIC 3086, Australia
[*]Correspondence and requests for materials should be addressed to: juncheng.e@xfel.eu, adrian.mancuso@diamond.ac.uk
[†]On leave from the Institute of Physics, Czech Academy of Sciences, Na Slovance 2, 182 21 Prague 8, Czech Republic

Diffraction patterns with Poisson noise

The diffraction patterns (Fig. S1) used for orientation recovery are calculated from a hydrated 2NIP protein after radiation damage with random orientation. We applied Poisson noise to the diffraction patterns before orientation recovery.

Temporal distribution of the diffraction intensity within a pulse

As mentioned in the main manuscript, the contribution of diffraction intensities from a sample structure snapshot can vary depending on the incident intensity. The simulated diffraction intensities for each X-ray pulse are the sum of the scattering intensities calculated for each 2.6 fs time interval. The scattering intensity for each time interval is the product of the square modulus of the evolved structure factor and the incident X-ray intensity in that time interval. The resulting scattering intensity temporal profile in the case of Pulse 1 (Fig. 5 in the main manuscript) is shown in Fig. S2.

EMC reconstruction results with/without inelastic scattering

The set of EMC reconstruction results of diffraction patterns (with or without inelastic scattering) from atomic structures after radiation damage is plotted in Fig. S3. There is no significant difference in the range of $q \leq 0.08$ Å^{-1} observed between the central XY slices with/without inelastic scattering, while the speckles are slightly blurrier in the range of $q \geq 0.1$ Å^{-1} for the slices with inelastic scattering than those without inelastic scattering.

The self-disconcurrence of the orientation recovery without inelastic scattering is plotted as the orange line in Fig. S4. Its value is in between the values of the self-OD without radiation damage (blue line) and that with both radiation damage and inelastic scattering (red line). Apparently, the inelastic scattering deteriorates the orientation recovery in addition to the effect of the radiation damage and thus should not be neglected.

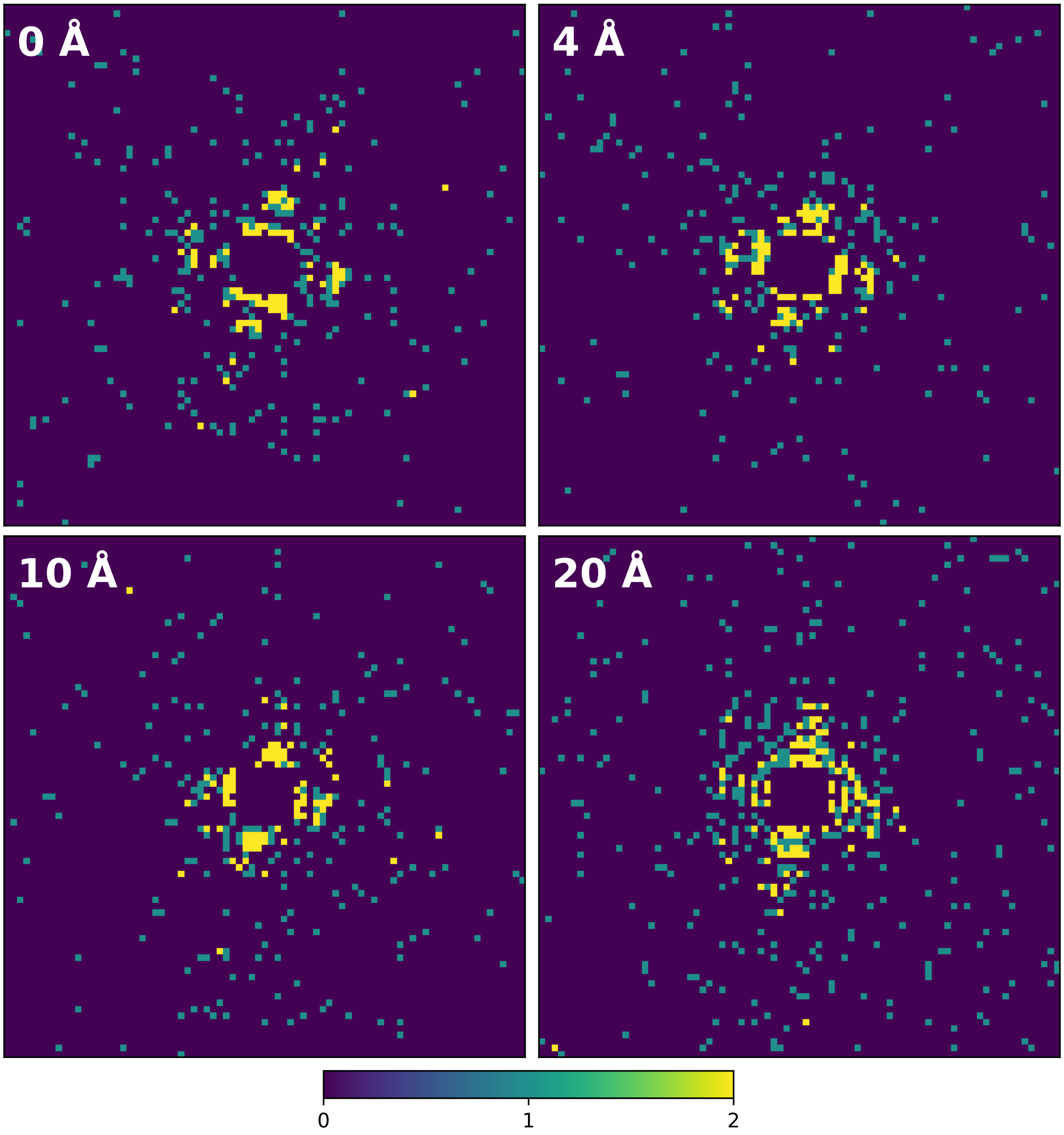

Figure S1. Typical simulated diffraction patterns with Poisson noise from a hydrated 2NIP protein endured radiation damage with various water layer thicknesses. $q = 0.14$ Å^{-1} at the edge of the diffraction patterns. The color map represents the number of photons.

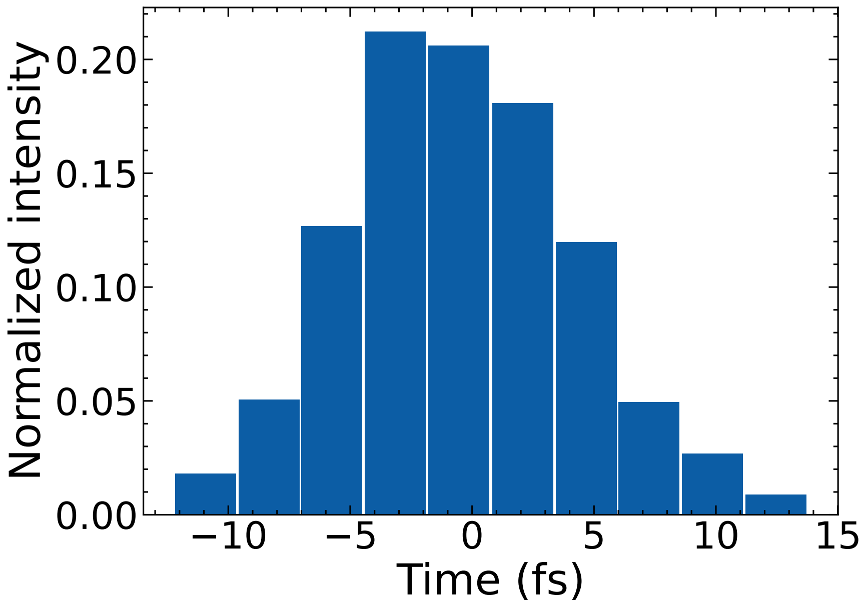

Figure S2. Temporal distribution of the diffraction intensity within a pulse. The intensity is binned with an interval of 2.6 fs, and normalized so that the sum of the intensities is 1.

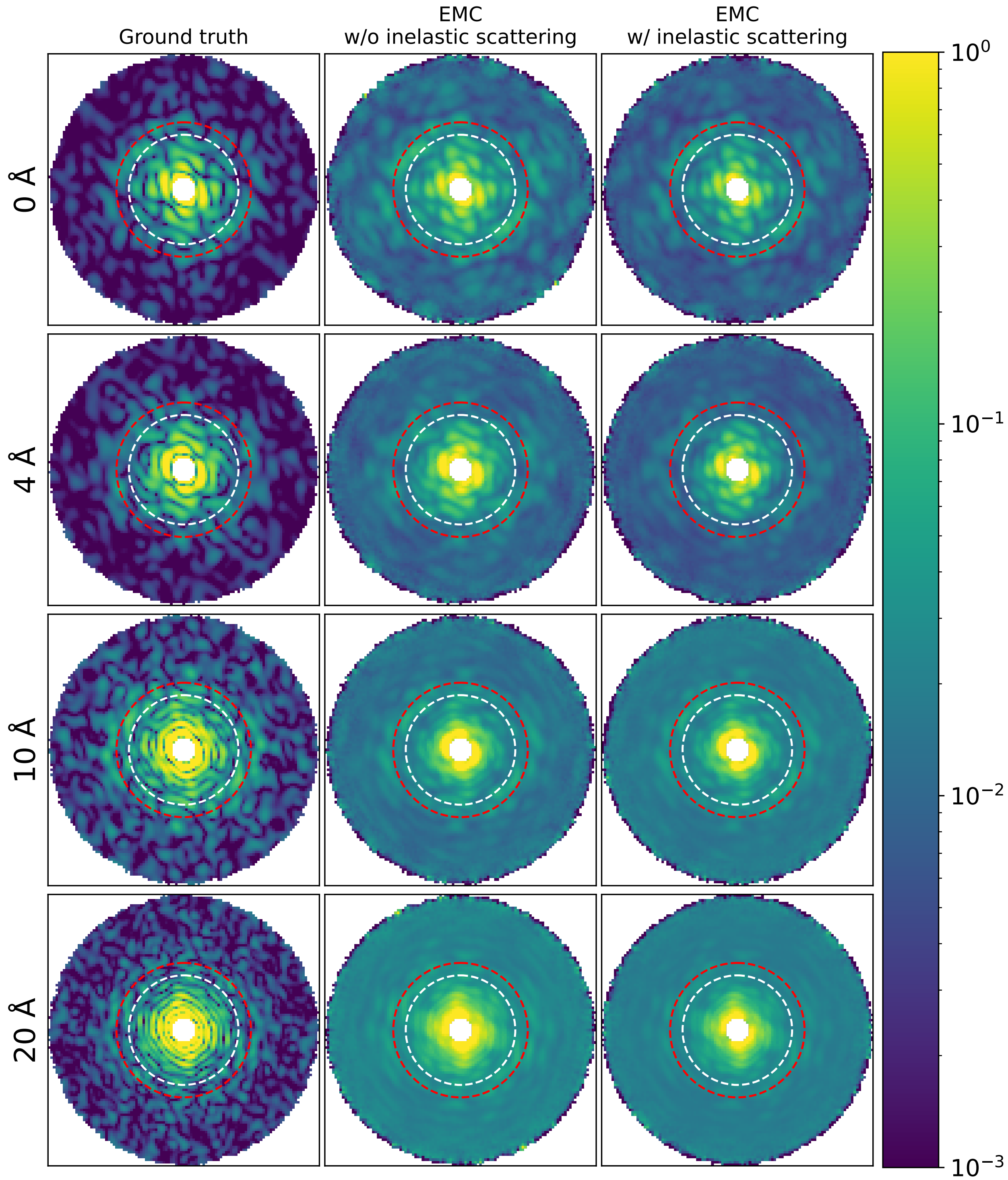

Figure S3. Central XY slices of the ground truth reciprocal spaces, and the reciprocal spaces reconstructed from diffraction patterns including or without inelastic scattering, recorded from radiation-damaged hydrated sample at various water layer thicknesses. The white and red dashed circles are at $q = 0.08$ Å^{-1} and $q = 0.10$ Å^{-1}, respectively. The q value at the edge is 0.2 Å^{-1}. The color map represents intensity and is plotted in arbitrary units.

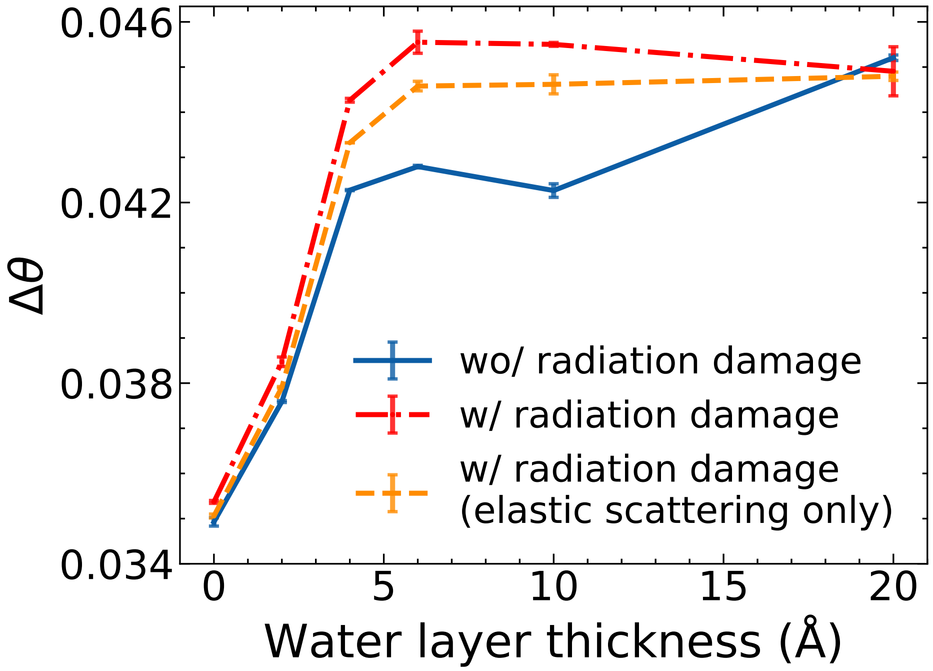

Figure S4. The self-disconcurrence of the orientation recovery with/without radiation damage as a function of water layer thicknesses. Each point on the curves is averaged over 3 independently reconstructed reciprocal space volumes, and its error bar is the standard deviation from the mean $\Delta\theta$. A lower value of $\Delta\theta$ indicates a better orientation recovery quality.
