# Supplementary figures and images for: Water layer and radiation damage effects on the orientation recovery of proteins in single-particle imaging at an X-ray free-electron laser
Source: Sci Rep. 2023 Sep 29;13:16359. doi: 10.1038/s41598-023-43298-1 (PMC10541445; doi:10.1038/s41598-023-43298-1)

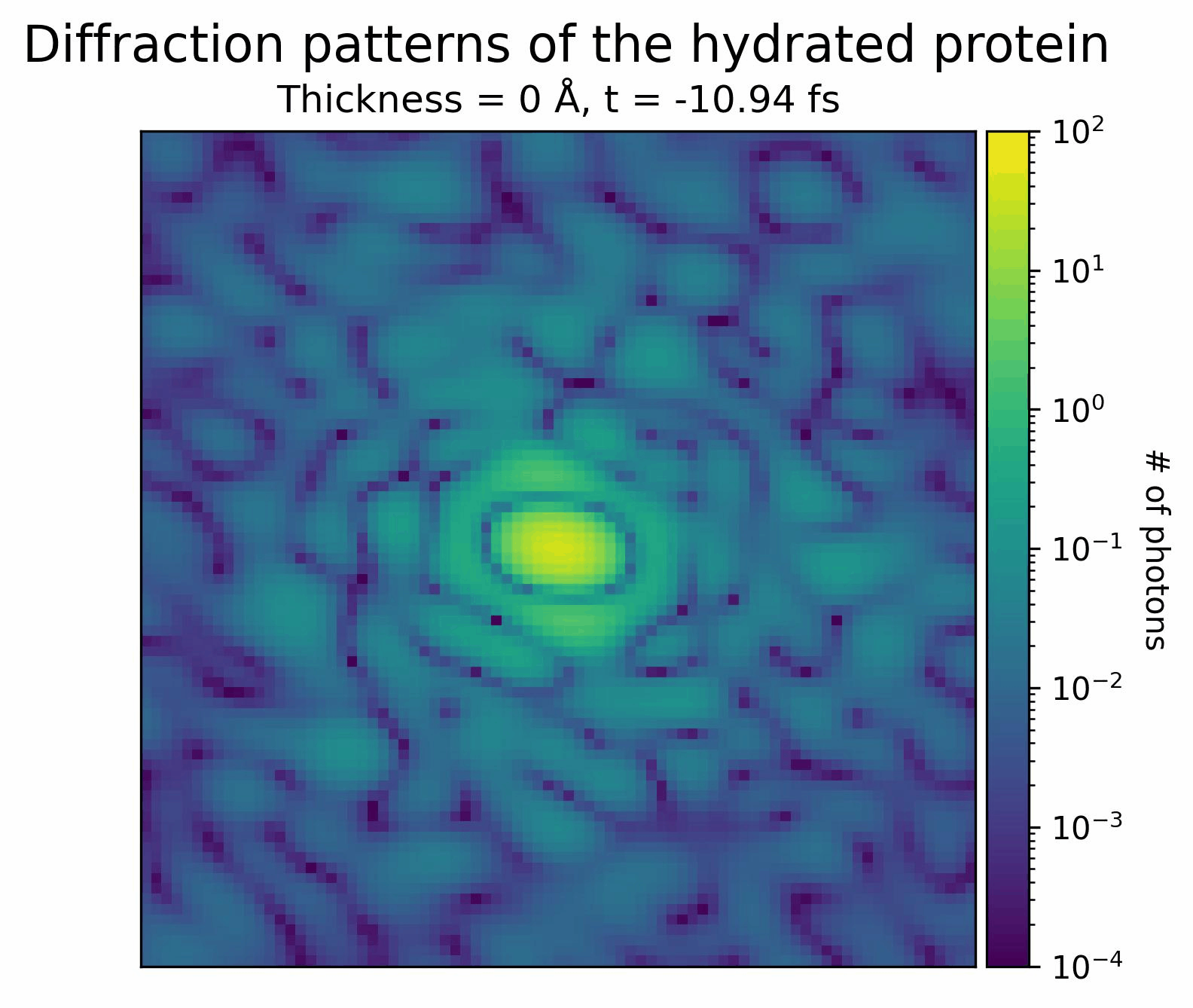

Supplement: Supplementary file 2 — Supplementary Information 2. [file 41598_2023_43298_MOESM2_ESM.gif]

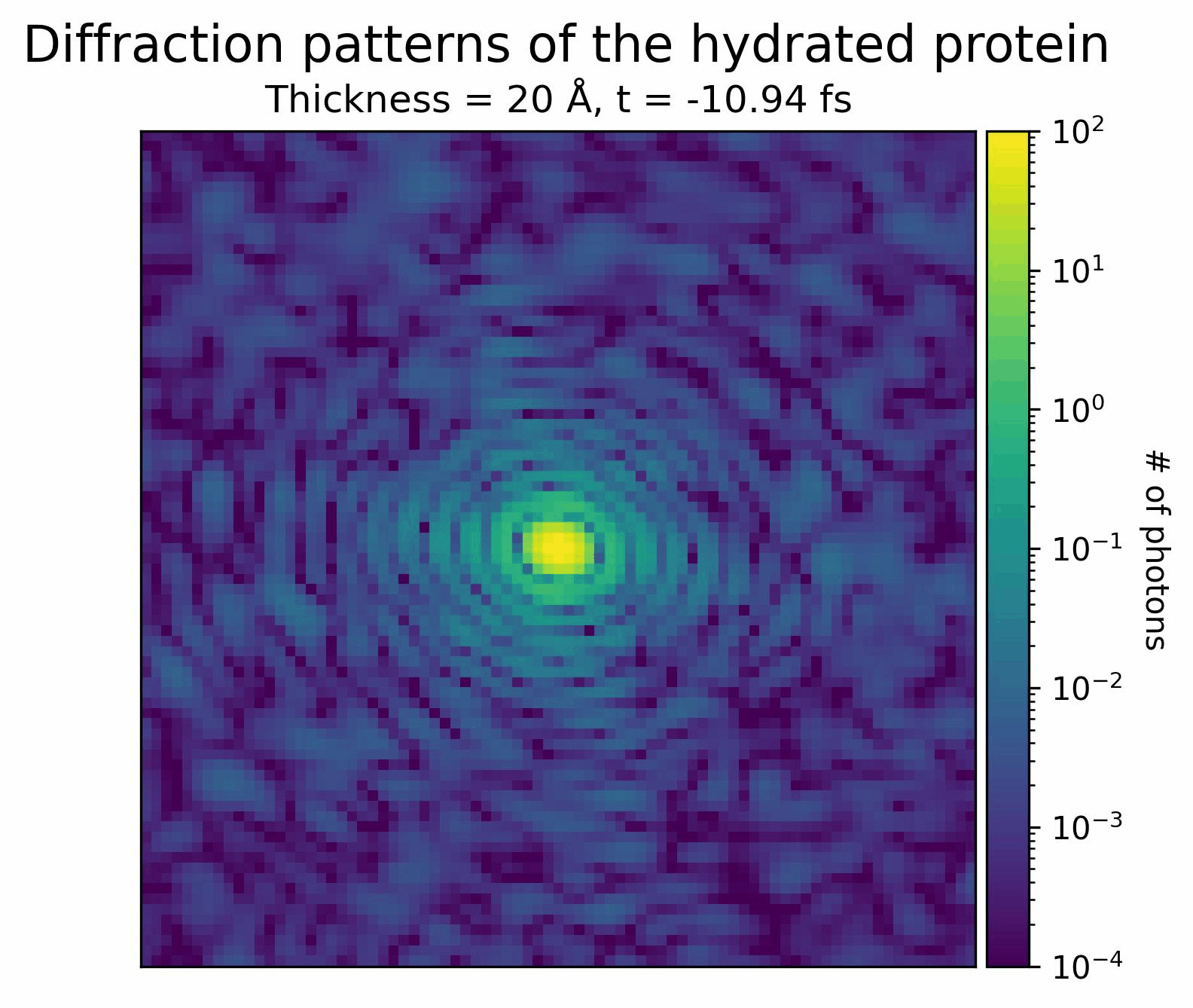

Supplement: Supplementary file 3 — Supplementary Information 3. [file 41598_2023_43298_MOESM3_ESM.gif]
